# Army Medical Intelligence—Official

**Published:** 1864-09

**Authors:** 


### ARMY MEDICAL INTELLIGENCE—OFFICIAL.

### SURGEON-GENERAL AND MEDICAL OFFICERS ON DUTY IN HIS OFFICE.

| | |
|---|---|
| S. P. Moore | Surgeon-General C. S. Army. |
| C. H. Smith | Surgeon C. S. Army. |
| Thos. H. Williams | " " " |
| F. Sorrel | " " " |
| Chas. Brewer | " P. A. C. S. |
| Herman Baer | " " " |

### MEDICAL DIRECTORS IN THE FIELD.

| | |
|---|---|
| Surgeon L. Guild | Army Northern Virginia. |
| " H. McGuire | Ewell's Corps, Army Northern Va. |
| " J. S. D. Cullen | Longstreet's Corps, " |
| " J. W. Powell | Hill's " " |
| " J. B. Fontaine | Cavalry " " |
| " John A. Hunter | Breckinridge's Command. |
| " R. L. Brodie | Beauregard's " |
| " T. L. Ozier, temp. | Charleston, S. C. |
| " A. J. Foard | Army of Tennessee. |
| " J. H. Erskine | Hindman's Corps, Army of Tennessee. |
| " A. L. Breysacker | Hardee's " " " |
| " P. B. Scott | Meridian, Miss. |
| " F. A. Stanford | Wheeler's Cavalry Corps. |
| " J. F. Heustis | Mobile, Ala. |
| " John M. Haden | Marshall, Texas. |
| " J. H. Berrien | Houston, " |
| " J. T. Darby | Stewart's Corps (Late Polk's) |
| " Will. Jennings | Morgan's Command. |

### OF HOSPITALS.

| | |
|---|---|
| Surgeon W. A. Carrington | Richmond, Va. |
| " F. A. Ramsey | Bristol, Tenn. |
| " P. E. Hines | Raleigh, N. C. |
| " N. S. Crowell | Charleston, S. C. |
| " S. H. Stout | Macon, Ga. |
| " S. A. Smith | Alexandria, La. |
| " J. F. Heustis | Mobile, Ala. |
| " P. B. Scott | Meridian, Miss. |

### MEDICAL INSPECTORS IN THE FIELD.

| | |
|---|---|
| Surgeon W. D. Tucker | Department of Ala., Miss. and La. |
| " Samuel Choppin | " N. C. and South. Va. |
| " R. J. Breckinridge | " Army Northern Va. |
| " J. W. Breedlove | Western Virginia. |
| " E. N. Covey | Va., Tenn. and Ga., and Superintend't of Vaccination of Armies in these States. |
| " W. W. Anderson | N. C., S. C., Ala., Fla., La. and Miss., and Superintendent of Vaccination of Armies in these States. |

### OF HOSPITALS.

| | |
|---|---|
| Surgeon T. C. Madison | Petersburg, Va. |
| " F. Sorrel | Richmond, " |
| " E. S. Gaillard | Box 1150 Richmond, Va. |
| " R. A. Kinloch | Charleston, S. C. |
| " W. M. Brown | Morton, Miss. |
| " E. A. Flewellen | Army of Tennessee. |
| " J. H. Morton | Abingdon, Va. |

### ARMY MEDICAL BOARDS.

| | | |
|---|---|---|
| Surgeon A. N. Talley | President of Board | Richmond, Va. |
| " E. Geddings | " " | Charleston, S. C. |
| " W. M. Brown | " " | Gen. Hood's Headquarters. |
| " J. J. Gaenslen | " " | Gen. E. K. Smith's Headquarters |
| " —— Hooper | " " | Trans-Miss. Department. |

### PRINCIPAL HOSPITALS IN THE CONFEDERATE STATES.

### VIRGINIA.

| Hospital. | Location. | Medical Officer. |
|---|---|---|
| Chimborazo | Richmond | Surg. J. B. McCaw. |
| Camp Jackson | " | " F. W. Hancock. |
| " Winder | " | " A. G. Lane. |
| " Lee | " | " W. P. Palmer. |
| Howard's Grove | " | " T. M. Palmer. |
| Stuart | " | " R. A. Lewis. |
| Louisiana | " | " W. C. Nichol. |
| General, No. 9 | " | " J. J. Gravatt. |
| " " 13 | " | " H. T. Barton. |
| " " 21 | " | " G. W. Semple. |
| " " 24 | " | " O. F. Manson. |
| " | Liberty | " B. Blackford. |
| " | Huguenot Springs | " W. T. Walker. |
| " | Gordonsville | " B. M. Lebby. |
| " No. 1 | Lynchburg | " G. W. Thornhill. |
| " " 2 | " | " W. C. N. Randolph. |
| " " 3 | " | " T. H. Fisher. |
| Ladies' Relief | " | " W. C. Warren. |
| Pratt | " | " J. H. Murray. |
| Way | " | " A. C. Smith. |
| General | Farmville | " H. D. Taliaferro. |
| " | Danville | " J. F. Fauntleroy. |
| " | Staunton | " A. M. Fauntleroy. |
| Confederate States | Petersburg | |
| General | " | |
| South Carolina | " | " F. Peyre Porcher. |
| Poplar Lawn | " | " R. P. Page. |
| Wayside | " | " M. P. Scott. |
| Small Pox | " | C. F. Couch, A. A. Surg. |
| North Carolina | " | Surg. J. G. Brodnax. |
| Virginia | " | " J. H. Pottenger. |
| General | Pearisburg | " T. Creigh. |
| " | Charlottesville | " J. L. Cabell. |
| " | Montgomery Sp'gs | " J. L. Woodville. |
| " | Emory | " J. B. Murfree. |
| " | Harrisonburg | " A. R. Meem. |
| Washington | Abingdon | " H. A. Blair. |
| Wayside | Burkesville | Dr. T. R. Blandy. |
| Breckinridge | Marion | Surg. R. D. Hamilton. |

### NORTH CAROLINA.

| Hospital. | Location. | Medical Officer. |
|---|---|---|
| Way, No. 5 | Wilmington | Surg. J. C. Walker. |
| General, No. 4 | " | " T. R. Micks. |
| " " 5 | " | " H. J. Macon. |
| Way, No. 7 | Tarboro' | Dr. J. H. Baker. |
| " " 1 | Weldon | Surg. H. H. Hunter. |
| " " 6 | Charlotte | " J. W. Ashby. |
| General, No. 11 | " | " " " |
| Gen. Military No. 2 | Wilson | " S. S. Satchwell. |
| Way | Goldsboro' | Dr. L. A. Stith. |
| General, No. 3 | " | " " W. A. Holt. |
| " " 7 | Raleigh | Surg. E. B. Haywood. |
| " " 8 | " | " H. G. Leigh. |
| Pettigrew | " | " E. B. Haywood. |
| Sorrel | Ashville | " W. L. Hilliard. |
| General, No. 9 | Salisbury | As. Sur. J. M. Fauntleroy. |
| " " 10 | " | Surg. J. W. Hall. |
| Way, No. 3 | " | " J. W. Hall. |
| General, No. 6 | Fayetteville | " " B. F. Fessenden. |
| General, No. 1 | Kettrell Springs | " H. F. Butt. |
| " " 12 | Greensboro' | " W. H. Moore. |
| Way, No. 2 | " | " E. B. Holland. |

[TO BE CONTINUED.]

